# Elucidating VSMC phenotypic transition mechanisms to bridge insights into cardiovascular disease implications

**DOI:** 10.3389/fcvm.2024.1400780

**Published:** 2024-05-13

**Authors:** Yuning Xin, Zipei Zhang, Shan Lv, Shan Xu, Aidong Liu, Hongyu Li, Pengfei Li, Huize Han, Yinghui Liu

**Affiliations:** ^1^ Traditional Chinese Medicine, Changchun University of Chinese Medicine, Changchun, China; ^2^ Traditional Chinese Medicine, The Affiliated Hospital of Changchun University of Chinese Medicine, Changchun, China; ^3^ Traditional Chinese Medicine, The Affiliated Hospital to Changchun University of Traditional Chinese Medicine, Changchun, China; ^4^ Traditional Chinese Medicine, The Third Affiliated Hospital of Changchun University of Chinese Medicine, Changchun, China

**Keywords:** vascular smooth muscle cells, phenotypic transition, cardiovascular diseases, atherosclerosis, molecular mechanisms

## Abstract

Cardiovascular diseases (CVD) are the leading cause of death worldwide, despite advances in understanding cardiovascular health. Significant barriers still exist in effectively preventing and managing these diseases. Vascular smooth muscle cells (VSMCs) are crucial for maintaining vascular integrity and can switch between contractile and synthetic functions in response to stimuli such as hypoxia and inflammation. These transformations play a pivotal role in the progression of cardiovascular diseases, facilitating vascular modifications and disease advancement. This article synthesizes the current understanding of the mechanisms and signaling pathways regulating VSMC phenotypic transitions, highlighting their potential as therapeutic targets in cardiovascular disease interventions.

## Graphical Abstract

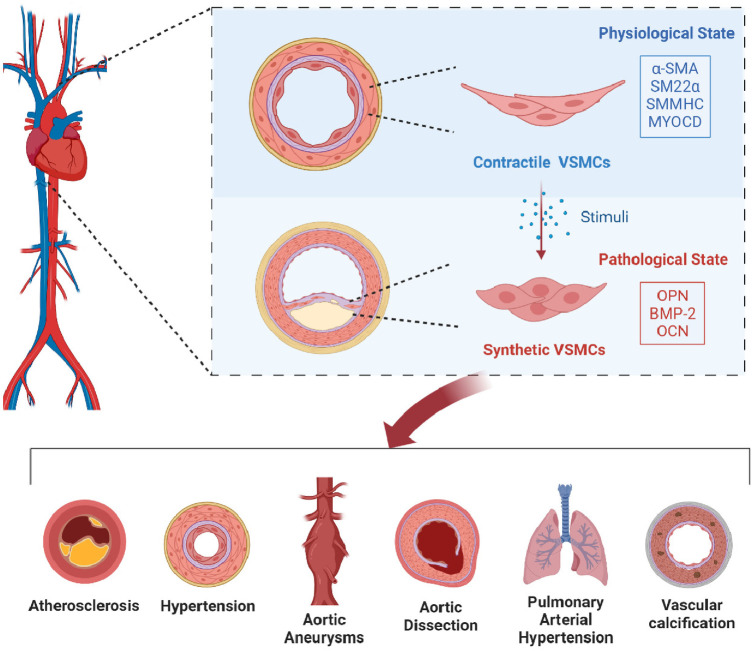

## Introduction

Cardiovascular diseases (CVD) represent a formidable challenge to global public health due to their extensive prevalence, serious health implications, and significant mortality rates (1). The burden of CVD is escalating, projected to become the leading financial strain in healthcare by 2035 (2). Particularly in China, the “2022 China Cardiovascular Diseases Report” (3) underscores a critical situation, with CVD surpassing cancer as the leading cause of mortality, fueled by a rising incidence and mortality rates. This is compounded by factors such as the rapid aging of the population and prevalent unhealthy lifestyles, placing a vast number of individuals at an elevated risk for CVD and amplifying the national health dilemma (4). Remarkably, CVD remains the chief cause of death in both urban and rural areas. In 2020, CVD was responsible for 48.00% of rural and 45.86% of urban fatalities, accounting for nearly half of all deaths (5). With approximately 330 million people in China affected by CVD, the urgency for enhanced prevention and treatment options is clear.

Recent research highlights the critical role of phenotypic transitions in vascular smooth muscle cells (VSMCs) in the progression of various CVDs (6). VSMCs, located in the middle layer of blood vessels, are essential for maintaining vascular tone, blood pressure regulation, and consistent blood flow, thereby ensuring vascular health (7). These cells are notably versatile, capable of changing their function in response to stress factors like hypoxia and inflammation, transitioning from a contractile to a secretory state (8). In this altered state, they release inflammatory cytokines, metalloproteinases, and other substances that contribute to the development of conditions such as atherosclerosis and pulmonary arterial hypertension (9). Targeting the prevention of VSMC phenotypic transition to preserve their contractile ability offers a promising strategy to slow disease progression. This article explores the mechanisms controlling VSMC phenotypic transition and its impact on cardiovascular health, establishing a foundation for therapeutic interventions targeting this process.

### Functions and phenotypic transition characteristics of VSMCs

The medial layer of blood vessels, consisting of VSMCs and the extracellular matrix (ECM), is essential for structural support and acts as the main layer for vascular contraction and dilation (10). VSMCs are notable for their ability to undergo non-terminal differentiation, exhibiting considerable potential for dedifferentiation and adaptability. This enables them to display two main phenotypes: the contractile and the synthetic (or secretory) (11). This adaptability is influenced by the dynamic interactions between VSMCs, the ECM, and various inflammatory mediators, which collectively guide the VSMCs’ phenotypic transitions.

In their default state, VSMCs exhibit a specialized contractile phenotype, characterized by a spindle-shaped appearance, abundant myofilaments, and a high proficiency in contraction, with minimal DNA synthesis activity and limited ECM production. This mature state is marked by elevated levels of essential contractile proteins such as alpha-smooth muscle actin (α-SMA), smooth muscle protein 22-alpha (SM22α), smooth muscle myosin heavy chain (SMMHC/SM-MYH), and myocardin (MYOCD) (12, 13), which are crucial for vascular contraction capabilities. The contractile properties of VSMCs, enhanced by their rich myofibrillar content, are pivotal in regulating vascular tension through periodic contraction and relaxation, thus contributing significantly to the mechanical functions of the vascular wall (14). The ECM supports this process by providing structural support and influencing VSMC behavior, phenotype, and function through its diverse components and structural properties, ensuring vascular integrity.

The synthetic phenotype of VSMCs is typically observed in developing embryos’ vascular structures or in diseased vessels, characterized by a flattened shape, reduced filament presence, decreased expression of contractile proteins, and increased expression of synthetic markers like osteopontin (OPN), bone morphogenetic protein 2 (BMP-2), and osteocalcin (OCN) (15). These cells exhibit a metabolic shift known as the Warburg effect, enhancing their proliferation and survival capabilities while increasing their production of ECM-related proteins such as matrix metalloproteinases (MMPs), urokinase-type plasminogen activator (u-PA), and tissue-type plasminogen activator (t-PA) (16), which facilitate their migration.

The plasticity of VSMCs is akin to the body's wound healing response, where appropriate vascular repair following an injury can promote the healing of localized damage, maintain homeostasis and structural integrity, and restore normal functions. However, inadequate repair may lead to vascular imbalance, characterized by VSMC overgrowth at the injury site, excessive ECM and enzyme secretion, and ECM remodeling, ultimately compromising the structure and function of the blood vessel wall. VSMCs undergoing phenotypic transition are identified as the primary contributors to vascular media remodeling, accounting for 95% of the cells involved (17). The phenotypic shifts in VSMCs are associated with the degradation of the vascular wall's normal structure and function, potentially initiating a spectrum of CVDs. Therefore, investigating the phenotypic transitions of VSMCs and their regulatory mechanisms is of paramount importance for the prevention and treatment of cardiovascular diseases.

### Common mechanisms of VSMC phenotypic transition

**Figure 1:**
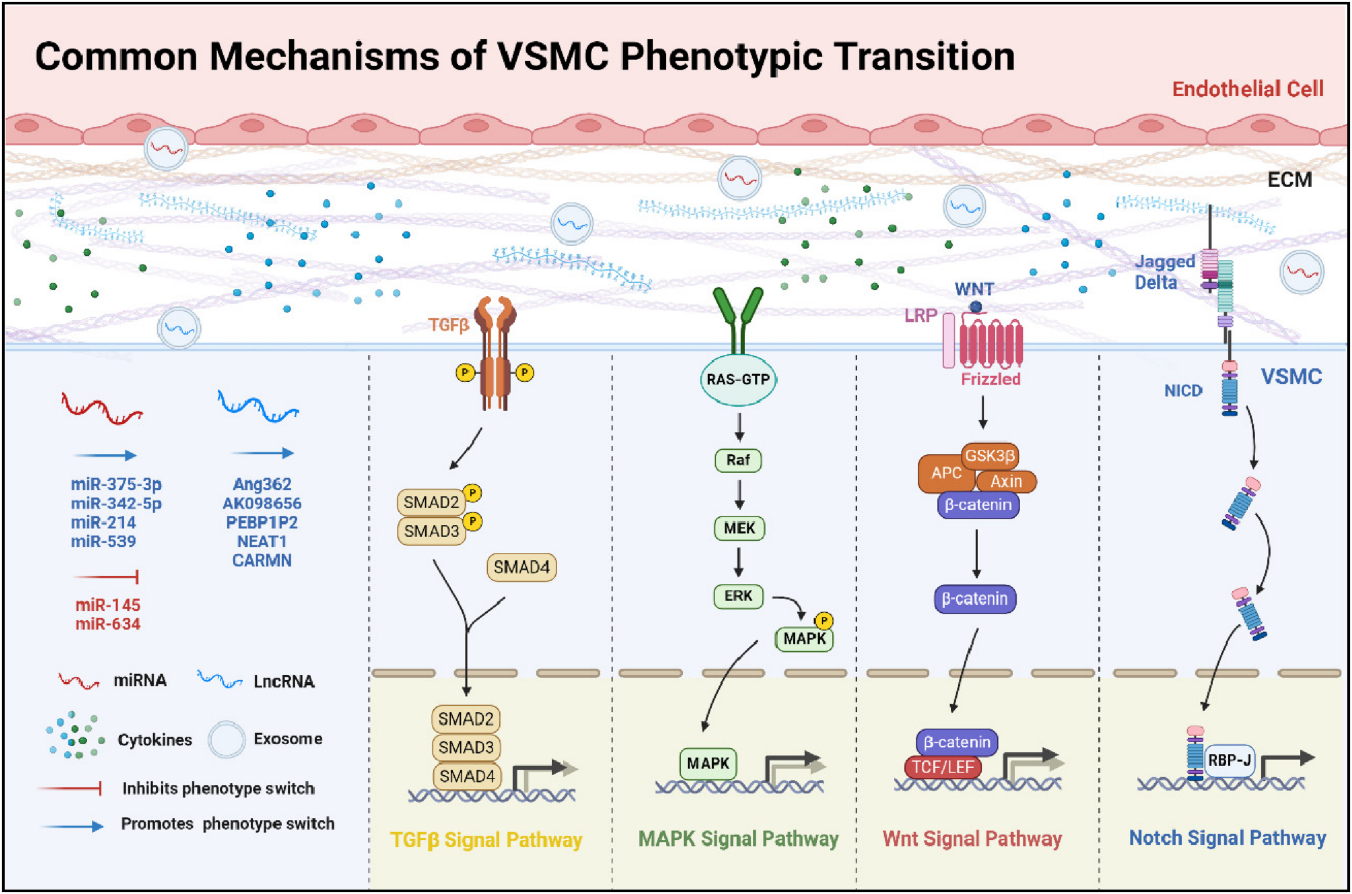
Common mechanisms of VSMC phenotypic transition. This diagram offers a detailed overview of the multifaceted mechanisms influencing the phenotypic transition of VSMCs. It delineates the interplay between pivotal signaling pathways: TGFβ, MAPK, Wnt, and Notch. The illustration details the modulatory impacts of miRNAs and LncRNAs, which can either inhibit or promote these transitions. Furthermore, it outlines the effects of ECM components and cytokines on this process. Collectively, the schematic underscores the complex network of interactions that modulate the functional dynamics and characteristics of VSMCs.

The phenotypic transformation of VSMCs is influenced by a variety of factors (Figure 1), including cytokines, signaling pathways, elements of the ECM, microRNAs, and biomechanical stresses (18). Understanding how these elements contribute to VSMC phenotypic transitions offers potential strategies for addressing cardiovascular diseases. This section focuses on the roles and mechanisms of these regulatory components and their signaling networks.

### Cytokines

Cytokines play a pivotal role in VSMC phenotypic transformation, especially under pathological conditions where there's an uptick in cytokine secretion. Among the key players are Platelet-Derived Growth Factor-BB (PDGF-BB) and Tumor Necrosis Factor-alpha (TNF-α) (19).

#### PDGF-BB

PDGF-BB is a peptide growth factor of the PDGF family, known for its synthesis in a wide range of cells, including VSMCs, fibroblasts, and macrophages (20). In healthy vasculature, PDGF-BB levels are typically low, but in conditions like atherosclerosis, its levels surge, driving VSMC proliferation and migration.

Zhou et al. (21) reveal how PDGF-BB influences VSMC phenotypic transitions by modulating levels of miR-214 and Pim-1, drawing an analogy to the epithelial-mesenchymal transition (EMT) process. EMT is a fundamental biological shift wherein epithelial cells develop mesenchymal characteristics such as increased mobility and invasiveness, playing a vital role in development, healing, and oncogenesis. Similarly, VSMCs respond to stimuli like PDGF-BB by shifting from a contractile to a synthetic phenotype, acquiring mesenchymal-like traits that enhance proliferation and migration. This similarity suggests that the molecular pathways of EMT may also be crucial in the phenotypic transformation of VSMCs, thereby impacting vascular remodeling and related diseases. The study also demonstrates that the PDGF-BB/PDGFR-β interaction also activates the ERK1/2 and JNK signaling pathways, resulting in shifts in VSMC phenotype. These shifts promote increased cellular growth and motility, contributing significantly to the pathogenesis of vascular diseases. Remarkably, the inhibition of the ERK1/2 pathway emerges as a potent strategy to counteract the phenotypic changes induced by PDGF-BB, highlighting the therapeutic potential of targeting such molecular pathways in vascular disease management. Further insights provided by Zhao et al. (22) augment our understanding of PDGF-BB's role in modulating VSMC phenotype, revealing its capacity to elevate DLEU2 and PCNA expression whilst concurrently suppressing α-SMA and CNN1 levels. These findings collectively underscore the integral role of PDGF-BB in orchestrating VSMC phenotypic transitions through a myriad of downstream effector pathways, highlighting its significance in the pathophysiology of vascular diseases.

#### TNF-α

TNF-α, released from damaged endothelial cells, initiates a cascade that accelerates VSMC proliferation and migration, fostering phenotypic shifts. Initially, TNF-α is shown to boost VSMC migration, with interventions targeting the Akt/AP-1 signaling pathway demonstrating efficacy in curbing these phenotypic transitions (23). Subsequently, TNF-α is observed to drive VSMC phenotypic alterations via the RhoA/cell cycle protein pathway, a process that can be mitigated by miR-145 through the downregulation of RhoA, effectively obstructing the TNF-α-induced phenotypic evolution (24). Moreover, exposure of human aortic endothelial cells to TNF-α triggers cytokine release, which in turn induces VSMC phenotypic modifications. These alterations lead to an upsurge in MMP-9 levels within VSMCs, culminating in the enhanced degradation of elastin and the accelerated progression of aortic aneurysms (25).

### Signaling pathways

The phenotypic transitions of VSMCs are critically influenced by various signaling pathways, including the TGF-β, Notch, MAPK, and Wnt pathways (26).

#### TGF-β signaling pathway

TGF-β signaling pathway is instrumental in controlling the phenotypic transitions of VSMCs (27). It functions by TGF-β binding to its receptors (TGF-βRII and TGF-βRI) on the VSMC surface, leading to the phosphorylation of Smad2 and Smad3 proteins (28). These phosphorylated Smad proteins then associate with Smad4 and translocate to the nucleus, where they enhance the expression of genes associated with the contractile phenotype of VSMCs (29). This process indicates that the TGF-β pathway generally promotes the maintenance of the contractile state in VSMCs, acting as a regulatory mechanism to prevent the transition towards a synthetic phenotype. However, the complexity of the TGF-β signaling pathway is highlighted by the role of Smad7, an inhibitory Smad that can interfere with this pathway. The presence of Smad7 reduces the phosphorylation of Smad3, which leads to a decreased expression of contractile markers in VSMCs (30), thereby facilitating a shift towards a synthetic or non-contractile phenotype.

Further research has shed light on the nuanced mechanisms through which TGF-β influences VSMC phenotypic transitions. For instance, TGF-β1 has been shown to affect the expression of pyruvate kinase M2 (PKM2) through the mTOR/c-Myc/PTBP-1/hnRNPA-1 pathway (31). This modulation of PKM2 expression enhances aerobic glycolysis in VSMCs, which is associated with their phenotypic transformation. This insight into the metabolic aspects of VSMC phenotypic transitions adds another layer to our understanding of how the TGF-β signaling pathway can influence VSMC behavior and function.

#### Notch signaling pathway

The Notch signaling pathway, a universally preserved mechanism, is pivotal in the cell-to-cell interactions and essential for the stability of VSMCs phenotypes. The contractile state of VSMCs is transcriptionally controlled by the SRF DNA-binding protein and the MYOCD transcriptional coactivator (32). Notch signaling enhances this regulatory mechanism by directly increasing MYOCD expression and independently activating specific structural genes in VSMCs during later developmental stages, underscoring its significance in vascular development and homeostasis.

Selective disruption of the RBP-J transcription factor, a key downstream component of the Notch pathway in VSMCs, induces a shift from a contractile to a synthetic phenotype. This phenotypic transition is associated with significant cardiac adjustments (33), highlighting the pathway's crucial role in cardiac and vascular health. Furthermore, targeted deletion of Jagged1 in endothelial cells leads to a concurrent decrease in Jagged1 and Notch3 expression in VSMCs (34). This reduction prompts the upregulation of extracellular matrix and adhesion molecules in VSMCs, contributing to their phenotypic transition and suggesting an intricate interplay between endothelial cells and VSMCs mediated by Notch signaling.

Building on these insights, experimental interventions in a mouse vein graft model further illustrate the therapeutic potential of modulating Notch signaling (35). The use of the γ-secretase inhibitor DAPT to suppress the Notch1 signaling pathway effectively slows the progression of neointimal hyperplasia in vein grafts. Moreover, soluble Jagged-1 has been demonstrated to block the Notch1/Hey2 signaling pathway, providing a promising therapeutic approach for treating neointimal restenosis in these grafts. These findings highlight the critical role of Notch signaling in preserving the structural integrity and functionality of blood vessels, with profound implications for understanding and treating various vascular diseases.

#### MAPK signaling pathway

MAPK pathway plays a critical role in VSMC phenotypic transitions, involving a series of serine/threonine protein kinases such as ERK1/2, JNK, and p38 MAPK. These kinases are central to conveying external signals into the cell, influencing a variety of cellular processes, including inflammation, proliferation, and differentiation (36). The interplay between these processes and VSMC phenotypic states is complex and significant for vascular health.

Research by Liu et al. (37) highlights the protective effect of inhibiting MAPK pathway activation on the preservation of the contractile phenotype in VSMCs. This suggests that MAPK pathway activity can drive VSMCs away from their contractile, quiescent state towards a more active, synthetic state. The activation of p38 MAPK, in particular, has been identified as a key factor in this phenotypic transition, especially in response to stimuli such as PDGF. This transition is critical in the context of vascular diseases, where the balance between VSMC phenotypes influences disease progression and outcomes (38).

Further insights from Zhang et al. (39) demonstrate how the peptide Aβ1-40 can disturb the phenotypic balance in VSMCs by increasing inflammatory markers like IL-1β and TNF-α, and altering the expression of phenotypic proteins. Specifically, Aβ1-40 leads to a decrease in α-SMA levels, indicative of the contractile phenotype, and an increase in OPN and KLF4 levels, associated with the synthetic phenotype. Moreover, Aβ1-40 enhances the phosphorylation of key MAPK proteins in VSMCs, including p38 MAPK, ERK1/2, and JNK. The use of specific MAPK inhibitors has been shown to significantly reduce IL-1β and TNF-α levels, highlighting the role of Aβ1-40 in promoting inflammation and phenotypic transitions in VSMCs through MAPK pathway activation.

#### Wnt signaling pathway

The Wnt signaling pathway, with its dual branches of classical (β-catenin-dependent) and non-classical (β-catenin-independent) signaling, plays a vital role in the regulation of VSMC phenotypic transformations, significantly influencing the development and progression of cardiovascular diseases (40). The classical pathway involves Wnt proteins binding to a receptor complex, leading to the stabilization and nuclear translocation of β-catenin, which then activates genes related to cell proliferation, migration, differentiation, and apoptosis. These processes are crucial for both cardiovascular development and the manifestation of various cardiovascular diseases. In contrast, the non-classical Wnt/Ca2 + pathway is associated with vascular calcification, illustrating the diverse impacts of Wnt signaling on cardiovascular health. This pathway's implications extend to various aspects of cardiovascular pathology, including the regulation of cholesterol levels and the maintenance of cardiovascular system homeostasis (41).

Recent studies have shed light on the interplay between the TGF-β/Smad3 and classical Wnt/β-catenin pathways in the context of vascular injury and repair, particularly in the formation of neointima following atherosclerosis. Elevated TGF-β/Smad3 expression post-injury has been shown to activate the classical Wnt pathway through the β-catenin mechanism, promoting VSMC transformation (42). Additionally, research into the role of podocan, a novel protein, in cardiovascular disease progression has revealed its significant impact on VSMC behavior. Overexpression of podocan in human VSMCs has been found to inhibit their migration and proliferation by targeting the Wnt-TCF pathway, providing further insight into the complex regulatory networks that govern cardiovascular pathologies (43).

### Extracellular matrix (ECM)

ECM is a sophisticated network composed of collagen, elastin, glycoproteins, and proteoglycans, providing essential structural support and influencing cellular behavior (44). Variations within this milieu can influence the phenotype of VSMCs in diverse ways.

One such ECM protein, Cartilage Oligomeric Matrix Protein (COMP), is critical in maintaining the contractile phenotype of VSMCs. High levels of COMP can alter VSMC structure, reduce the expression of contractile markers like SM22, α-SMA, and CNN1, and effectively inhibit the PDGF-BB-induced phenotypic shift in VSMCs. This illustrates the intricate relationship between ECM components and VSMC phenotype regulation (45).

The influence of collagen on VSMC phenotype is complex and depends on its form. VSMCs cultured on monomeric collagen I exhibit increased levels of Vascular Cell Adhesion Molecule (VCAM-1) and undergo phenotypic transition. In contrast, polymerized type I collagen appears to mitigate this transition by upregulating p27Kip1 and p21Cip1, which helps to restrict VSMC migration and proliferation (46). Further research by Mao et al. (47) has highlighted the role of nidogen-2, a basement membrane component, in maintaining VSMC contractile state. Nidogen-2 enhances the interaction between Jagged1 and Notch3, thereby promoting Notch signaling activity within VSMCs. The absence of nidogen-2 leads to reduced Notch signaling, inducing a shift in VSMC phenotype and contributing to neointimal hyperplasia.

Elastin is another crucial ECM component that impacts VSMC maturation, phenotype, and proliferation. A deficiency in elastin has been shown to activate the mTOR signaling pathway in VSMCs, prompting a phenotypic transition (48). Moreover, enzymes involved in ECM degradation, such as MMP-2, also play a role in VSMC phenotypic modulation. MMP-2, in particular, can induce a phenotypic shift in VSMCs by reducing CNN1 levels and increasing ERK1/2 phosphorylation, thereby stimulating cell growth (49). These findings underscore the pivotal influence of ECM components on the regulatory mechanisms of VSMC phenotype.

### Biomechanical forces

VSMCs are dynamically responsive to various mechanical stresses, including cyclic stretch, pressure, and shear stress, which significantly influence their phenotypic transitions. The effects of mechanical stress on VSMCs are multifaceted, impacting their contractility, inflammatory response, and proliferative capacity, thus playing a crucial role in vascular health and disease.

Research by Wang et al. (50) demonstrated that prolonged exposure to high tension (>10%) leads to a reduction in contractile markers like CNN1, SM22, and α-SMA in human umbilical artery VSMCs, alongside an increase in inflammatory markers such as IL-8, IL-6, IL1β, VCAM-1, and ICAM-1. This indicates that elevated mechanical tension can promote a phenotypic shift in VSMCs towards a more synthetic, less contractile state. Similar observations were made by Jensen LF et al. in rat VSMCs under comparable tension conditions, further supporting the notion that mechanical stress can drive VSMC phenotypic changes (51). Zhou and colleagues explored the molecular mechanisms underlying these changes, revealing that increased mechanical tension activates the ROCK/JNK/SP1 signaling pathway in VSMCs. This pathway inhibits the transcription of Mitofusin 2 (MFN2), leading to elevated PFK1 protein levels and increased cellular glycolysis by preventing PFK1's ubiquitination and degradation. This metabolic shift, reminiscent of the Warburg effect observed in cancer cells, supports increased cellular proliferation and migration, contributing to the phenotypic transition of VSMCs (52).

The ECM's role in mediating the effects of mechanical stress on VSMCs is also significant. Variations in ECM composition and stiffness can induce changes in external pressure on VSMCs, promoting phenotypic alterations. Studies using polyelectrolyte hydrogels of different densities have shown that substrate rigidity can influence VSMC phenotype, with physiological levels of stiffness supporting the maintenance of the contractile phenotype (53, 54). Shear stress, particularly in the context of endothelial layer disruption as seen in atherosclerotic lesion ruptures, exposes VSMCs directly to blood flow, inducing phenotypic changes. Laminar shear stress has been shown to activate mechanisms that counteract PDGF-BB-induced VSMC proliferation, such as enhanced NOS activity and AMPK phosphorylation (55). Similarly, exposure to shear stress can promote a contractile phenotype in VSMCs through mechanisms involving ROCK1 levels and MYPT1 and MLC activation, highlighting the complex interplay between mechanical forces and VSMC phenotypic state (56). Furthermore, the activation of mechanosensitive pathways, such as those involving Piezo1 channels, in response to shear stress can lead to calcium influx, contributing to VSMC phenotypic shifts and increased proliferation. This can result in vascular wall thickening and reduced lumen size, illustrating the profound impact of mechanical stimuli on vascular structure and function (57).

### Non-coding RNAs

Non-coding RNAs play a pivotal role in the complex regulatory networks governing cellular processes, among which microRNAs (miRNAs) and long non-coding RNAs (lncRNAs) are particularly noteworthy for their roles in vascular biology. miRNAs, short RNA sequences approximately 22 nucleotides in length, function by binding to complementary sequences in the 3’ untranslated regions (3’ UTRs) of target mRNAs, thereby inhibiting their translation or leading to mRNA degradation. In contrast, lncRNAs are longer transcripts that do not code for proteins but can regulate gene expression through a variety of mechanisms, including chromatin modification, transcriptional interference, and acting as molecular decoys.

The introduction and widespread use of high-throughput sequencing technologies have dramatically enhanced our understanding of the role of these non-coding RNAs, such as miRNAs and lncRNAs, in regulating VSMC phenotypic transitions. These regulatory RNAs play significant roles in modulating VSMC behavior from contractile to synthetic phenotypes and vice versa.

#### miRNAs

miRNAs have emerged as key players in this regulatory landscape. Various miRNAs, including miR-375-3p, miR-214, and miR-342-5p, have been identified to promote a synthetic phenotype in VSMCs, while miR-145 and miR-634 help maintain their contractile state. These miRNAs exert their effects by targeting specific genes that influence VSMC behavior. For example, miR-214 can lead to a reduction in contractile protein expression by increasing Smad7 levels and decreasing Smad3 phosphorylation, thus facilitating a shift in VSMC phenotype (58). Similarly, miR-375-3p targets PDK1, promoting phenotypic changes in VSMCs (59).

On the other hand, miR-145, abundant in healthy VSMCs, decreases in aortic dissection cases. Its overexpression can boost contractile markers in VSMCs, inhibiting phenotypic transitions. Additionally, (60). miR-634, by targeting the 3'UTR of Wnt4, prevents its expression in HASMCs and the subsequent nuclear translocation of β-catenin, effectively blocking the Wnt4/β-catenin pathway and inhibiting VSMC phenotypic transformation (61). These insights highlight the critical regulatory roles of miRNAs in VSMC phenotypic transitions, offering potential targets for therapeutic intervention in vascular diseases characterized by abnormal VSMC behavior.

#### LncRNAs

LncRNAs are crucial in the complex regulation of VSMCs, facilitating their adaptive shifts between contractile and synthetic phenotypes in response to various stimuli. These lncRNAs modulate VSMC behavior through diverse mechanisms, influencing cellular proliferation, migration, and phenotype. For example, lnc-Ang362 plays a significant role in the response of VSMCs to Angiotensin II (Ang II), promoting cell proliferation and migration by increasing miR-221 and miR-222 levels, activating the NF*κ*B pathway, and enhancing phosphorylation of key proteins in this signaling cascade (62). Similarly, the presence of oxidized Low-Density Lipoprotein (ox-LDL) elevates lncRNA LINC00341 expression, which also encourages VSMC proliferation and migration (63).

LncRNA AK098656 is another key player that induces VSMC phenotypic transitions by enhancing matrix protein production and accelerating the breakdown of contractile proteins through direct interactions with cellular structural components (64). In contrast, lncRNA PEBP1P2 has an inhibitory effect on VSMC proliferation, migration, and phenotypic shifts, highlighting the diversity in lncRNA functions within VSMC biology (65).

Moreover, lncRNA NEAT1 has been shown to bolster VSMC proliferation and migration primarily through its association with WD repeat domain 5B, which deters cell differentiation and influences phenotypic transitions (66). Additionally, Cardiac Mesoderm Enhancer-Associated Non-Coding RNA (CARMN), a lncRNA uniquely expressed in smooth muscle cells and integral to cardiac differentiation, exhibits notably diminished expression in vascular disease contexts in both humans and mice. CARMN significantly uplifts the activity of the key transcriptional coactivator Myocardin, thus preserving the contractile phenotype of VSMCs, underlining the diverse and complex roles lncRNAs play in VSMC biology and vascular disease mechanisms. The results highlight the intricate role of non-coding RNAs in VSMCs phenotypic transitions, emphasizing the necessity for additional research to clarify their precise functions and underlying mechanisms (67).

### Exosomes (EXO)

Exosomes, tiny vesicles ranging from 40 to 160 nanometers, are key players in cell-to-cell communication, encapsulating a variety of functional biomolecules. In vascular systems, VECs release exosomes loaded with biomolecules like miRNAs, lncRNAs, and proteins, which have profound effects on the behavior of VSMCs (68).

Under normal physiological conditions, VECs secrete exosomes containing miR-143 and miR-145, crucial for maintaining the contractile function of VSMCs. However, following vascular injury, the levels of these miRNAs in exosomes decrease, contributing to a phenotypic shift in VSMCs towards a more synthetic, less contractile state. For instance, miR-539 can transfer from VECs to VSMCs via exosomes, promoting a shift towards a proliferative phenotype in VSMCs (69).

Moreover, exosomes carrying circHIPK3 from mouse aortic endothelial cells can induce VSMC proliferation under high glucose conditions and prevent apoptosis via the miR-106a-5p/Foxo1/Vcam1 pathway (70). Similarly, VEC-derived exosomes containing miR-342-5p can influence not only cardiomyocyte behavior but also VSMC phenotype, showcasing the versatility of exosomal cargo in modulating cellular functions across different cell types (71).

Beyond miRNAs, VEC-derived exosomes also carry other biomolecules like lncRNAs and proteins, further influencing VSMC behavior. Activation of specific pathways can lead to changes in the contents of these exosomes, such as a reduction in TET2 protein levels, which in turn can enhance VSMC proliferation and migration, facilitating a shift to a synthetic phenotype (72). Oxidized LDL can also alter the cargo of exosomes, with lncRNA LINC01005 carried in such exosomes inducing phenotypic changes in VSMCs via the miR-128-3p–KLF4 signaling axis (73). These examples highlight the critical role of endothelial cell-derived exosomes and their diverse contents in regulating VSMC behavior and phenotype.

### Other factors

Glycolysis plays a crucial role in VSMC phenotypic adaptation. For instance, in pulmonary arterial smooth muscle cells (PASMCs) affected by PAH, there's a significant increase in the levels of Aldehyde Dehydrogenase 1 Family Member A3 (ALDH1A3). Disrupting ALDH1A3 expression in VSMCs can halt the progression of hypoxia-driven PAH by reducing glycolytic activity, thus preventing the phenotypic shift in VSMCs (74). Similarly, Pyruvate Kinase M2 (PKM2), a key enzyme in glycolysis, is upregulated in VSMCs during the progression of AS. Inhibiting PKM2 can decrease VSMC proliferation and migration, slowing AS development. These findings suggest that targeting glycolytic pathways in VSMCs could be a viable approach to managing AS and PAH (75).

Histone modifications, including methylation, acetylation, and phosphorylation, are critical for the regulation of gene expression. The methylation mark H3K4me2, found on genes specific to smooth muscle, plays a pivotal role in maintaining VSMC phenotype. This epigenetic marker remains present in the promoter regions of smooth muscle genes in both differentiated and dedifferentiated states, highlighting its importance in VSMC identity. The removal of H3K4me2 methylation, via the demethylase LSD1, leads to a loss of VSMC contractility and identity, promoting a phenotypic shift (76). Histone Deacetylase 6 (HDAC6) is another key player in the epigenetic regulation of VSMC phenotype. Inhibiting HDAC6 can activate the transcription factor SRF and increase the levels of contractile proteins such as α-SMA and MRTF-A, preventing VSMC phenotypic transition. These epigenetic mechanisms underscore the complexity of VSMC phenotype regulation and offer potential therapeutic targets for vascular diseases characterized by aberrant VSMC behavior (77).

### The role of VSMC phenotypic transition in common cardiovascular diseases

Synthetic phenotype VSMCs contribute significantly to cardiovascular disease by overproducing extracellular matrix components and pro-inflammatory cytokines, leading to inflammation and endothelial dysfunction. Their increased proliferation also causes vessel wall thickening and narrowing, playing a pivotal role in vascular pathology.

### Atherosclerosis (as)

AS is a chronic inflammatory disease characterized by the formation of plaques within the arterial intima (17). Compared to VSMCs in healthy vessels, those in AS exhibit reduced expression of contractile molecules such as SM22 and α-SMA, while the expression of secretory markers like OPN and MMPs is increased (78). Genetic tracing studies have shown that about 70% of VSMCs in AS plaques originate from the phenotypic transformation, proliferation, and migration of medial VSMCs, thereby contributing to plaque formation (79).

Recent research has emphatically highlighted the critical role of the NLRP3 inflammasome in the progression of atherosclerosis. Notably, studies (80) utilizing TET2-deficient mouse models have demonstrated that activation of the NLRP3 inflammasome significantly accelerates atherosclerosis by enhancing inflammatory responses and facilitating plaque formation. Furthermore, it has been observed that nicotine can activate the NLRP3 inflammasome in VSMCs, thereby promoting the formation and increasing the instability of atherosclerotic plaques (81). Particularly, in ApoE^−/−^ mouse models, nicotine treatment elevates IL-1β levels in the serum and aorta. This elevation further stimulates the NLRP3 inflammasome, thereby hastening the development of atherosclerotic plaques.

Recent advances in atherosclerosis research have illuminated the molecular mechanisms behind VSMC transformations, crucial for plaque development. Floriana MF (82) found that during the phenotypic transformation of VSMCs, the expression of DOT1l, a histone H3 lysine 79 (H3K79) methyltransferase, is upregulated. DOT1l and its induced histone mark, H3K79me2, can directly regulate NF-kB transcription, inducing VSMC phenotypic transformation and promoting the expression of CCL5 and CXCL10, thus facilitating the progression of AS. Additionally, under AS conditions, the interaction between immune cells and VSMCs can regulate VSMC phenotype. Macrophages, which are abundant in AS plaques, have their M1 polarization, inflammatory factor secretion, and RAGE/TLR4/FOXC2 signaling reduced by inhibiting TLR4, which in turn suppresses Dll4 expression in macrophages. These macrophages can then promote VSMC phenotypic transformation through the Dll4/Notch pathway upon direct contact with VSMCs in the aorta (83). Chen and colleagues discovered that in ApoE^−/−^ mice with atherosclerosis, the expression of T cell death-associated gene 8 (Tdag8) is significantly increased. Tdag8 mediates VSMC phenotypic transformation through the cAMP/PKA signaling pathway, thereby promoting the development of AS (84).

Recent studies have also highlighted the role of VSMC phenotypic transformation in plaque stability. The myocardin-SRF complex, a central component of VSMC phenotype regulation and a coactivator for most VSMC contractile genes, has been implicated in this process. Bennett and others found that myocardin^+/−^ mice on an ApoE knockout background showed increased AS severity and macrophage accumulation compared to myocardin^+/+^ littermates, with VSMCs being the sole vascular cells expressing myocardin (85). Furthermore, KLF4 directly interacts with SRF, disrupting the myocardin-SRF complex and inhibiting the expression of VSMC contractile genes, thereby mediating VSMC phenotypic transformation (86). Genetic lineage tracing has demonstrated that selective knockout of KLF4 in VSMCs leads to a significant reduction in lesion size, increased fibrous cap thickness, and enhanced plaque stability in advanced AS lesions (87).

### Hypertension

Hypertension is defined by a narrowing of vascular diameter resulting from enhanced vasoconstriction and arterial remodeling, which collectively lead to higher vascular resistance. This condition is driven by a variety of factors, including the Renin-Angiotensin-Aldosterone System (RAAS), sympathetic nervous system activity, immune responses, and oxidative stress (88).

In the context of hypertension-induced vascular remodeling, VSMCs are critically involved. The calcium-sensing receptor (CaSR) within VSMCs, serving as an extracellular Ca2 + receptor, is instrumental in modulating vascular dilation through the regulation of calcium ion levels both locally and systemically (89). The importance of CaSR in VSMCs for regulating blood pressure has been underscored by various research studies, though results have varied. For instance, research conducted by Yamamura and associates (90) found that activating CaSR significantly raises blood pressure in mice models of pulmonary hypertension. In a different study, Wonneberger and team (91) discovered CaSR mRNA in VSMCs of guinea pig primary radial arteries, observing that calcium ions and specific CaSR agonists induce vascular contraction. Furthermore, employing the allosteric inhibitor NPS2143 in rat models has been observed to increase blood pressure, possibly by interacting with the RAAS system (92).

The immune and inflammatory responses play a pivotal role in the phenotypic alteration of VSMCs. Ge (93) observed a notable rise in M1-type macrophages in hypertensive patients with vascular calcification, suggesting a defense mechanism against this calcification. The presence of M2-type macrophages is elevated by the combined effects of serum OPG and OPN, with OPN also capable of reducing M1-type macrophage expression. Pro-inflammatory cytokines, such as TNF-α and IL-1β, produced by M1-type macrophages, alongside ROS, contribute to the worsening of vascular endothelial function and VSMCs (94), thus aggravating hypertension and subsequent organ damage. The NLRP3 inflammasome, crucial for innate immunity and cardiovascular damage sensing, significantly influences vascular inflammation and VSMCs’ phenotypic changes in hypertension (95). Gan's study indicates (96) that Ang II enhances NLRP3 inflammasome activation and pro-inflammatory cytokine release, driving the inflammatory response in myocardial fibrosis. Conversely, the removal of the NLRP3 gene lessens Ang II's effects on VSMCs phenotypic shifts and vascular restructuring. Krishnan's findings (97) suggest that the NLRP3 inflammasome inhibitor MCC950 lowers blood pressure in hypertensive rodents, and alleviates renal inflammation and fibrosis. Additionally, Animal studies have demonstrated that the AK098656 transgenic rat model accurately reflects the initial pathogenic stages seen in primary hypertension patients, presenting a new avenue for investigating hypertension's origins and potential treatment options (64).

### Aortic pathologies

Aortic Aneurysms (AA) and Aortic Dissection (AD) represent two critical aspects of aortic pathology, both characterized by structural alterations in the aortic wall, albeit with distinct clinical manifestations. AAs, particularly abdominal aortic aneurysms (AAA), are primarily characterized by localized or widespread expansions of the arterial wall, stemming from arterial damage or disease, leading to a compromised vascular integrity. The development of AAs is primarily attributed to the breakdown of extracellular matrix elements and arterial medial layer components like elastin, culminating in diminished vascular wall tension and compromised vascular integrity (98).

Research has consistently shown a decline in the expression of VSMCs contractile proteins, such as α-SMA and SM-MHC, in models of thoracic and abdominal aortic aneurysms, as well as Marfan syndrome (MFS), indicating a pivotal role of VSMCs in the pathogenesis of AA (99). This is further corroborated by research demonstrating that targeted deletion of TGFβ-R2 in VSMCs not only triggers their transformation into various cell types, thereby facilitating the formation of aortic aneurysms (100), but also induces phenotypic shifts in AAAs’ VSMCs towards proliferative, synthetic variants, serving as a protective mechanism (101). Furthermore, Marc et al. put forward a theory in their study on AA suggesting that the proliferation of VSMCs might offset the heightened cell mortality observed during AA evolution. By employing lineage tracing in mice, they observed a particular group of VSMCs exhibiting clonal growth within the thoracic and abdominal aorta's medial layer following Ang II infusion. This expansion spanned from the aortic wall into the adventitia and the newly formed false lumen (102). Hence, Marc et al. proposed that these VSMCs-derived cells could serve a healing function during the progression from aneurysm formation to aortic dissection.

Aortic Dissection (AD) occurs when blood infiltrates the aortic media, forming a false lumen (103). The VSMC phenotype regulation plays a significant role in AD, where factors leading to VSMC dedifferentiation may exacerbate the condition. Bendall and associates, utilizing an endothelial cell-specific Nox2 transgenic mouse model, found that VECs from these mice trigger VSMCs activation via CypA release, resulting in Erk1/2 phosphorylation within VSMCs (104). Genetic factors like mutations in the FBN1 gene in MFS can lead to compromised aortic integrity and influence VSMC phenotypic shifts (105), while non-genetic factors such as hypertension and atherosclerosis also contribute to AD through alterations in VSMC phenotype (106). In AD mouse models, ALDH2 intervention decelerates dissection by modulating VSMC phenotype shifts (107), with human data suggesting ALDH2's protective role against thoracic dissections. Conversely, ANXA1 downregulation exacerbates dissection through VSMC phenotypic alterations, whereas ANXA1 mimetics confer vascular benefits (108).

In both AA and AD, the phenotypic transformation of VSMCs plays a central role, where these cells undergo changes that impair the aorta's structural integrity and function. The release of MMPs by transformed VSMCs in AAA leads to the accelerated breakdown of the extracellular matrix (109), and similar mechanisms are observed in AD where altered VSMC phenotype contributes to the progression of the dissection (106). Additionally, the involvement of specific pathways like the pannexin-1 (Panx-1)/P2Y2 receptor pathway in VSMC activation indicates shared molecular targets that could be explored for therapeutic interventions in both conditions (110).

In conclusion, the interconnected nature of Aortic Aneurysms and Aortic Dissection underscores the importance of VSMCs and their phenotypic plasticity in the pathogenesis of aortic diseases. Understanding the shared and distinct mechanisms underlying these conditions is crucial for developing targeted therapies that address the complex interplay of factors contributing to aortic pathology.

### Pulmonary arterial hypertension (PAH)

PAH is recognized for its high morbidity and mortality, largely attributed to extensive pulmonary vascular remodeling. The damage to blood vessels triggers a crucial shift in PASMCs from their contractile nature to a synthetic one, setting off the pathological vessel changes (111). This shift, coupled with the migration and proliferation of PASMCs from the vessel's medial layer to its inner lining, results in significant thickening of the vessel walls. Furthermore, PASMCs extend their reach to the smaller distal pulmonary arterioles, proliferating and contributing to the formation of plexiform lesions. These lesions, consisting of narrowed arteriolar lumens, manifest as clusters, webs, or nodules, exacerbating the condition. While VSMCs, fibroblasts, and circulating progenitor cells, collectively termed smooth muscle-like cells, are involved in PAH development, lineage tracing has pinpointed VSMC phenotype-transformed cells as the primary agents of arteriole remodeling (112).

In Congenital Heart Disease-related PAH (CHD-PAH) models (113), induced by surgically creating a systemic-to-pulmonary shunt, progressive pathologic alterations in pulmonary arteries are evident. These changes commence with the proliferation of ECs and their disarray, which progressively intensifies. PASMCs then start to mimic ECs, proliferating upwards against the internal elastic lamina and thus narrowing the vascular lumen. Over time, marked proliferation and hypertrophy of PASMCs manifest, characterized by fatty changes, disorganized structures, and uneven lumen narrowing, culminating in near-total occlusion. The transformation and altered functionality of PASMCs are central to PAH pathogenesis.

Research has identified a significant increase in the glycolytic enzyme α-enolase (ENO1) within PASMCs of both PAH patients and Hypoxic Pulmonary Hypertension (HPH) animal models (114). This elevation of ENO1 is unique to PASMCs, with no similar increase observed in pulmonary arterial endothelial cells or fibroblasts. Inhibiting ENO1 gene expression has been shown to curtail PASMC proliferation and their transition to a less differentiated state, whereas ENO1 overexpression amplifies their synthetic characteristics via the AMP-activated protein kinase (AMPK)-AKT signaling pathway. Additionally, Morris et al. (115) have demonstrated an upsurge in NOTCH3 expression within PAH patient PASMCs, with the intensity of PAH in humans and rodents directly linked to NOTCH3 concentrations in lung tissues. Activation of NOTCH signaling is associated with the enhancement of PASMCs’ synthetic phenotype, and therapeutics targeting NOTCH pathways have shown promise in mitigating PAH-related vascular abnormalities.

The study led by Cordes and colleagues (116) represents a significant leap forward in our understanding of the miRNA that govern the differentiation of VSMCs. MiR-145 stands out due to its unique binding sites for SRF, a transcription factor that, when allied with myocardin and myocardin-related transcription factors (MRTFs), significantly boosts the expression of miR-145. This elevation in miR-145 levels is not merely a biochemical event but a pivotal moment in the regulatory saga of VSMC phenotypic determination, with myocardin playing a starring role. The activation of miR-145 transcription, spurred by TGF-β through myocardin, is a testament to the dynamic interplay between these molecular entities, steering significant phenotypic transformations within VSMCs. This rich tapestry of molecular interactions is further exemplified by experimental findings from miR-143/145-deficient mouse models (117). These models reveal a striking shift in the VSMC landscape, marked by a decrease in contractile phenotype cells and a concurrent rise in synthetic phenotype cells within the pulmonary arteries, when compared to their wild-type counterparts. This phenotypic shift sheds light on the potential role of VSMC transformation in the pathogenesis of PAH, thereby opening new avenues for understanding and potentially treating this complex disease.

### Vascular calcification (Vc)

VC is a prevalent pathological manifestation in diseases involving vascular remodeling. Traditionally viewed as a passive accumulation of calcium salts associated with aging, recent research now identifies VC as an active, regulatable process similar to bone development or cartilage formation. During this process, VSMCs undergo a phenotypic transformation, generating matrix vesicles that facilitate the deposition of calcium phosphate within vessel walls, thus forming primary calcification lesions. Elevated extracellular phosphate levels are recognized as primary promoters of this osteogenic transformation. Specifically, high phosphate environments lead to decreased XBP1u levels (118), resulting in the upregulation of osteogenic markers such as Runx2 and Msx2 that exacerbate VC.

At the molecular level, genes such as Alkbh1 and IDO1 significantly influence VC. Research by Liu et al. (119) highlights that modulation of Alkbh1, through either overexpression or inhibition, significantly impacts VC progression. Enhanced activity of Alkbh1 aggravates VC by increasing the demethylation of 6 mA, whereas its inhibition slows the progression. Alkbh1 also activates BMP2 transcription by facilitating Oct-4 binding to the BMP2 promoter, thus promoting VSMC phenotypic transformation. Moreover, IDO1, which is implicated in tryptophan metabolism, plays a pivotal role in the phenotypic modulation of VSMCs. Studies in animal models have shown that the absence of IDO1 exacerbates calcific lesion formation, indicating that IDO1-mediated metabolites advance RUNX2 ubiquitination through the aryl hydrocarbon receptor-dependent pathway (120). Furthermore, PDGF-BB has been identified as a pivotal player in vascular calcification (121). Research in mouse models demonstrates that serum levels of PDGF-BB increase with age, directly correlating with an increase in VC(122). These findings are supported by *in vitro* assays, which confirm that PDGF-BB can dose-dependently accelerate the calcification process (123).

Additionally, hyperhomocysteinemia (HHcy), known to be associated with cardiovascular diseases, has been shown to induce vascular calcification by promoting the expression of the transcription factor KLF4. This in turn upregulates RUNX2, enhancing the osteogenic potential of vascular cells (124). Research by Zhu and colleagues (125) has confirmed a correlation between elevated homocysteine levels and the incidence of VC, suggesting its utility as a biomarker for the progression and onset of calcification in both coronary and extracoronary arteries. Another significant factor in the development of VC is Transcription Factor 21 (TCF21), which is highly expressed in arterial atherosclerotic plaques in both humans and mice. TCF21 facilitates VC through the enhancement of IL-6 expression and subsequent activation of the STAT3 pathway, illustrating a complex interplay of inflammatory responses in calcification processes (126). Finally, the role of Stromal Interaction Molecule 1 (STIM1), located on the endoplasmic reticulum membrane, is crucial in regulating intracellular calcium balance. A deficiency in STIM1 leads to disrupted calcium homeostasis, activating calcium signaling pathways in VSMCs (127). This increase in endoplasmic reticulum (ER) stress promotes the osteogenic transformation and calcification of VSMCs, highlighting a key aspect of cellular stress response in the pathology of vascular calcification.

### Summary and expectation

Despite significant advances in our understanding of cardiac health, formidable challenges in preventing and managing cardiovascular diseases persist. VSMCs and their dynamic phenotypic changes play a pivotal role in cardiovascular pathologies. This review critically examines how phenotypic transformations of VSMCs contribute to the development of pathological states and evaluates the latest research directions in this field.

The initial adaptive response of VSMC phenotypic transformation is aimed at repairing vascular damage and accommodating physiological changes. However, prolonged responses can lead to detrimental shifts, precipitating severe conditions such as atherosclerosis and aortic dissection. Key signaling pathways such as TGF-β, mediated through Smad proteins, generally sustain the contractile phenotype but can shift toward a synthetic phenotype influenced by factors like Smad7 (30). The Notch (33), Wnt (41), and MAPK (37) pathways also critically maintain VSMC phenotype stability and regulate cellular behavior in response to external stimuli.

The regulatory role of microRNAs, particularly miR-145, is essential in maintaining the contractile phenotype, with its downregulation linked to increased proliferation and synthetic phenotype shifts, commonly seen in hypertension (60). Exploring the regulation of these microRNAs could pave the way for novel strategies aimed at preventing or reversing pathological vascular remodeling. Additionally, The influence of biomechanical forces on VSMC phenotype underscores the complexity of environmental impacts on cellular behavior. Mechanical stress, such as that induced by hypertension, prompts VSMCs to transition to a synthetic phenotype, leading to vascular stiffness and impaired function. Current research into how mechanical signals are converted into cellular responses offers promising pathways for developing treatments that target these mechanotransductive pathways to preserve vascular health.

Glycolysis, significantly influenced by enzymes like ALDH1A3 and PKM2, plays a crucial role in VSMC phenotypic transformations associated with diseases like PAH (74) and AS (75). The immune and inflammatory responses, evidenced by increases in M1-type macrophages and pro-inflammatory cytokines (94), exacerbate vascular dysfunction, highlighting the interplay between metabolic activities and immune responses in vascular pathologies

Advances in single-cell genomics and high-throughput screening technologies have unveiled new therapeutic targets and revolutionized our understanding of VSMC phenotypic changes in CVD. However, significant gaps remain in fully validating these transformations and their clinical implications. Future research should focus on elucidating the complex molecular mechanisms controlling VSMC behavior, with an emphasis on maintaining phenotype stability and preventing pathological remodeling. Developing new drugs that specifically target these pathways holds promise for treating or potentially preventing the progression of cardiovascular diseases. Identifying early pathological changes and relevant biomarkers is crucial for advancing targeted therapies and improving clinical outcomes.
